# Are there specific clinical characteristics associated with physician’s treatment choices in COPD?

**DOI:** 10.1186/s12931-019-1156-1

**Published:** 2019-08-20

**Authors:** Nicolas Roche, Anestis Antoniadis, David Hess, Pei Zhi Li, Eric Kelkel, Sylvie Leroy, Christophe Pison, Pierre-Régis Burgel, Bernard Aguilaniu, COLIBRI COPD Research Group

**Affiliations:** 1 Service de Pneumologie, Hôpitaux Universitaires Paris Centre, Hôpital Cochin, AP-HP and Université Paris Descartes, Sorbonne Paris Cité, 75014 Paris, France; 2 Laboratoire LJK, Département de statistiques, Université Grenoble Alpes, Grenoble, France; 3 Programme Colibri-Pneumo, ACCPP (Association pour la Complémentarité des Connaissances et des Pratiques de la Pneumologie), Grenoble, France; 4 Respiratory Epidemiology and Clinical Research Unit, Montreal Chest Institute, McGill University, Montreal, Canada; 5 Service de pneumologie, Pôle médecines spécialisées et cancérologie, Centre hospitalier général, Chambéry, France; 6 Department of Pulmonary Medicine and Oncology, CHU de Nice, University Hospital Federation OncoAge, Nice, France; 7 CNRS UMR 7275 - Institut de Pharmacologie Moléculaire et Cellulaire, Université de Nice Sophia Antipolis, Nice, France; 8 Service Hospitalier Universitaire Pneumologie Physiologie, Pôle Thorax et Vaisseaux, Centre Hospitalier Universitaire Grenoble Alpes, Inserm 1055, Université Grenoble Alpes, Grenoble, France; 9 Université Grenoble Alpes and Programme Colibri-Pneumo (aCCPP), Grenoble, France; 10 Pneumologie et Soins Intensifs Respiratoires, Hôpital Cochin, 27, rue du Fbg St Jacques, 75014 Paris, France

**Keywords:** COPD, Treatment, Clinical impact, Exacerbations

## Abstract

**Background:**

The number of pharmacological agents and guidelines available for COPD has increased markedly but guidelines remain poorly followed. Understanding underlying clinical reasoning is challenging and could be informed by clinical characteristics associated with treatment prescriptions.

**Methods:**

To determine whether COPD treatment choices by respiratory physicians correspond to specific patients’ features, this study was performed in 1171 patients who had complete treatment and clinical characterisation data. Multiple statistical models were applied to explain five treatment categories: A: no COPD treatment or short-acting bronchodilator(s) only; B: one long-acting bronchodilator (beta2 agonist, LABA or anticholinergic agent, LAMA); C: LABA+LAMA; D: a LABA or LAMA + inhaled corticosteroid (ICS); E: triple therapy (LABA+LAMA+ICS).

**Results:**

Mean FEV1 was 60% predicted. Triple therapy was prescribed to 32.9% (treatment category E) of patients and 29.8% received a combination of two treatments (treatment categories C or D); ICS-containing regimen were present for 44% of patients altogether. Single or dual bronchodilation were less frequently used (treatment categories B and C: 19% each). While lung function was associated with all treatment decisions, exacerbation history, scores of clinical impact and gender were associated with the prescription of > 1 maintenance treatment. Statistical models could predict treatment decisions with a < 35% error rate.

**Conclusion:**

In COPD, contrary to what has been previously reported in some studies, treatment choices by respiratory physicians appear rather rational since they can be largely explained by the patients’ characteristics proposed to guide them in most recommendations.

**Electronic supplementary material:**

The online version of this article (10.1186/s12931-019-1156-1) contains supplementary material, which is available to authorized users.

## Background

During the last two decades, the number of pharmacological agents available for COPD has increased significantly, especially among inhaled treatments [1]. These remain the cornerstone of therapy for chronic bronchial diseases. However, the number of inhaled pharmacological classes present in treatment algorithms has not changed markedly [2], and changes have corresponded mostly to improvements in the pharmacokinetic profiles of molecules, the design of new inhalation devices [3] and the combination of pharmacological classes within the same device [4, 5]. Among oral drugs, the rank of theophylline and its derivatives in treatment strategies has markedly decreased while PDE4 inhibitors have been marketed in select indications, although their access to the market was denied in some countries [1]. Long-term macrolides have also been tested with some success to prevent exacerbations and now appear in guidelines [1]. The level of evidence of mucoactive agents has improved although some uncertainty remains [6].

In parallel, there has been a proliferation of guidelines on COPD care, produced at various levels ranging from global (i.e., Global initiative on chronic Obstructive Lung Disease, GOLD) [1] to continental (e.g., from the European Respiratory Society, ERS or American Thoracic Society, ATS), national or even more local [7]. One crucial issue in the current treatment paradigms is personalisation, as part of 4P (personalised, predictive, preventive and participatory) or precision medicine [8, 9]. Accordingly, a lot of research is ongoing to identify endotypes, i.e., biological mechanisms that can be identified using biomarkers and targeted by specific treatments, and are associated with one or more clinical phenotypes with specific evolutionary profile and/or response to treatments [8]. Regarding COPD, only two biomarkers are now considered in guidelines: one is alpha1 antitrypsin (AAT) status (AAT deficiency affects a small minority of emphysematous patients), the other, introduced very recently, is blood eosinophil count [1].

The main challenge for the next years or decades will be to develop new targeted treatment strategies based on deciphering disease’s heterogeneity and underlying pathophysiological mechanisms [10]. As mentioned above, awaiting this (r) evolution guidelines developers make constant efforts to propose up-to-date evidence-based treatment strategies designed to provide the right treatment to the right patient at the right moment, one definition of personalised medicine. Patients characteristics used to guide treatment decisions are dominated by symptoms (mostly dyspnea and/or health status, as well as chronic mucus production for some treatments, i.e. roflumilast), exacerbation history and lung function [1]. The way these variables and others (e.g., comorbidities, age, persistent smoking …) are associated with physicians’ treatment choices is not clearly known, although this understanding is crucial to maximise guidelines’ implementation in the real-life by field physicians, which is known to be disappointing [11–16].

In this context, the goal of this study was to identify COPD clinical characteristics associated with treatment choices made by respiratory physicians during real-life routine visits, using multiple explanatory statistical approaches.

## Material and methods

### The COLIBRI cohort: general design

The COLIBRI project has been described previously [17]. Its primary aim is to propose a standardised and structured web-based medical consultation. Participants are voluntary respiratory physicians in France. All entered data are stored in a secured central server certified for health data storage (OVH Healthcare, Claranet). The database has been authorised by the French national commission on personal data privacy (Commission Nationale de l’Informatique et des Libertés, CNIL, authorisation number # 2013–526) after a positive advice from the committee on health data management for research purposes (Comité Consultatif sur le Traitement de l’Information en matière de Recherche dans le domaine de la Santé, CCTIRS). The requirement for written consent was waived in this observational cohort study in accordance with French law. Patients provided oral consent following information by their physician. The project was launched in March, 2013 in the Rhône-Alpes French Region and was subsequently extended to other participants on the French territory. Altogether, at present the project comprises 145 respiratory physicians working in hospitals (78%) or private practices (22%). Among hospital-based physicians, 83 (73%) work in tertiary care university hospitals. All patients with a spirometry-confirmed physician diagnosis of COPD can enter the database.

### Data collection

The COLIBRI project collects data on treating physicians and patients. Physicians’ data include type of activity (university hospital, general hospital, private clinic, mixt), type of area of activity (town, rural). The main patients’ data include demographic and anthropometric characteristics, risk factors (smoking history, professional exposure, occupation), comorbidities, respiratory symptoms, exacerbation history, findings at physical examination, self-estimated time spent walking outside the home, modified Medical Research Council dyspnea scale (mMRC), Epworth Sleepiness Scale, COPD assessment test (CAT), Hospital Anxiety and Depression (HAD) scale, Disability Related to dyspnea COPD Tool (DIRECT) [18], lung function tests, arterial blood gases and pharmacological and non-pharmacological treatments.

### Treatment categorization

For the present analyses, treatments were categorized as follows:

- A category: no COPD treatment or short-acting (SA) bronchodilator(s) (beta2 agonist, SABA and/or anticholinergic, SAMA) only;
- B category: one long-acting (LA) bronchodilator (beta2 agonist, LABA or anticholinergic agent, LAMA);
- C category: LABA+LAMA;
- D category: one long-acting bronchodilator + inhaled corticosteroid (ICS);
- E category: triple therapy (LABA+LAMA+ICS).

Of note, the present analyses did not include any focus on oral COPD drugs since roflumilast is not available in France and theophylline derivatives are very infrequently used.

Treatment prescriptions were analysed for each GOLD ABCD category. The concordance with current guidelines was assessed by calculating the proportion of patients from A and B categories who received an ICS, the proportion of all patients who were not prescribed any short-acting rescue bronchodilator and the proportion of B, C and D categories who did not receive any long-acting bronchodilator.

### Statistical analysis

All analyses were performed using the R statistical software, version 3.2.4 and the SAS statistical software, version 9.2 (SAS Institute, Cary, NC, USA). Results were considered statistically significant when the probability of a type I error was below 5%.

The analyses were done with data from all individuals with complete records for age, height, weight, gender, FEV1, FVC, exacerbation history, comorbidities, mMRC, CAT, HAD and DIRECT scores, and pharmacological treatments. Continuous data are presented as means and standard deviations while categorical data are presented as percentages. The characteristics of the patients in the two populations, the one with complete records and the one with incomplete records, were compared with the non-paired independent samples Student’s t-test for continuous variables and Chi-2 or Fischer’s exact test for proportions.

To examine the relationship between patients’ prescribed treatments and their characteristics several statistical models have been applied in parallel to the observed data in order to identify in a reliable way the predominant subset of covariates that influence the therapeutic regimens.

In a first phase, the patients’ prescribed treatment were recoded as a dichotomous response, according to the following itemisations:

- A vs BCDE: no vs at least one maintenance treatment. Then patients with no maintenance treatments were excluded from the subsequent analyses.
- B vs CDE: one vs more than one maintenance treatment
- BC vs DE: without vs with ICS
- E vs BCD: triple therapy vs all other options

Then we applied an a priori defined strategy of multivariate analyses comprising multiple logistic regression, penalized multiple logistic regression, and a nonparametric technique based on an evolutionary algorithm for learning globally optimal classification and regression trees (see detailed explanations in the Additional file 1).

The set of explanatory variables, composed by a mixture of continuous and categorical variables and introduced in the models, contains the following: age, gender, HAD, CAT, DIRECT, FEV1, FVC, and exacerbation history. The results of the various tested methods (multiple logistic regression, penalized multiple logistic regression, and the nonparametric technique based on classification and regression trees) were compared in terms of their ability to PREDICT treatment prescription. To evaluate the performance of each model and to compare them efficiently, a random split of the data was performed into a training sample (*n* = 800) for learning and a test sample (*n* = 311) for validation. Using the validation sample allow a valid unbiased estimation of the true misclassification rate (probability of prediction error, PPE, corresponding to misclassification rate).

In a second phase, since in reality the prescribed therapeutic treatment is a multi-categorical variable, we have used a multinomial multiple logit regression for nominal multi-category responses and parameters glyphs to visualize the effect strengths by star plots, where one star collects all the parameters connected to each selected term (R-package EffectStars) [19].

Finally (third phase), to investigate and confirm, in a descriptive way, the impact of the predictors on the response variable and examine the degree of their correlation, we have also used a mosaic display which is appropriate for the analysis of multiway contingency tables [20]. More precisely, we have fitted a regression tree model to the data to predict the response’s status from a selected set of predictor variables and then, used the splits on the selected variables, to recode all variables as categorical before applying the mosaic methodology.

Ultimately, since there was a strong interaction between mMRC and DIRECT, it was decided to perform all the above-mentioned analyses of associations with the DIRECT first, then to repeat them with mMRC dyspnea grade, keeping or excluding the DIRECT.

## Results

### Patients

Among the 4140 patients in the COLIBRI database on April 3rd, 2017, 1171 had complete data for all the required variables and were included in the analyses. Their characteristics are described in Table 1 and compared with those of patients who could not be included in the analyses due to incomplete data. Patients with complete data comprised a slightly lower proportion of GOLD 4 and a slightly higher proportion of GOLD 3 category. Slightly less patients were on LTOT (long-term oxygen therapy) and CPAP (Continuous Positive Airway Pressure) but more patients were on NIV (Non Invasive Ventilation). A greater percentage of patients were at-risk of future exacerbations following the GOLD criteria. Some other statistically significant differences were found but their magnitude was of marginal clinical significance.

**Table 1:** Characteristics of the total COLIBRI population at the time of data extraction, and comparison between patients with or without complete data

Characteristics	Total		Complete data		Incomplete data		Overall *P* Value
	N	Mean ± SD /n (%)	N	Mean ± SD /n (%)	N	Mean ± SD /n (%)	
Age, in year	4140	65.9 ± 9.9	1171	65.2 ± 8.4	2969	66.1 ± 10.4	0.005*
Gender, male, n(%)	4146	2910 (70.2)	1171	811 (69.3)	2975	2099 (70.6)	0,411
BMI, kg/m^2^	3866	25.8 ± 5.9	1171	26.1 ± 5.9	2695	25.6 ± 6.0	0.044*
COPD GOLD stages, n(%)	3730		1171		2559		
GOLD I		745 (20.0)		241 (20.6)		504 (19.7)	0,53
GOLD II		1594 (42.7)		501 (42.8)		1093 (42.7)	0,967
GOLD III		883 (23.7)		294 (25.1)		589 (23.0)	0,163
GOLD IV		508 (13.6)		135 (11.5)		373 (14.6)	0.012*
VEMS, % predicted	3730	59.4 ± 22.8	1171	59.9 ± 22.1	2559	59.2 ± 23.0	0,293
CVF, % predicted	3670	85.1 ± 22.7	1171	87.7 ± 23.4	2499	83.9 ± 22.2	< 0.001*
Active smoker, n(%)	3829	1321 (34.5)	1158	449 (38.8)	2671	872 (32.6)	< 0.001*
Cigarette smoker pack-years	3614	43.3 ± 24.4	1132	45.6 ± 22.8	2482	42.3 ± 25.0	< 0.001*
mMRC Score	3231	1.9 ± 1.2	1171	1.7 ± 1.1	2060	1.9 ± 1.2	< 0.001*
mMRC Score ≥ 2, n (%)	3231	1839 (56.9)	1171	627 (53.5)	2060	1212 (58.8)	0.004*
HAD Score	1621	12.3 ± 7.2	1171	12.3 ± 7.1	450	12.5 ± 7.5	0,877
CAT score	1863	16.7 ± 7.7	1171	16.8 ± 7.7	692	16.6 ± 7.9	0,545
DIRECT score	1803	12.1 ± 7.7	1171	11.8 ± 7.6	632	12.6 ± 7.9	0,05
Treatment category	3642		1171		2471		
A		676 (18.6)		214 (18.3)		462 (18.7)	0,76
B		620 (17.0)		223 (19.0)		397 (16.1)	0.026*
C		534 (14.7)		217 (18.5)		317 (12.8)	< 0.001*
D		498 (13.7)		132 (11.3)		366 (14.8)	0.004*
E		1314 (36.1)		385 (32.9)		929 (37.6)	0.006*
LTOT, n(%)	4157	768 (18.5)	1171	170 (14.5)	2986	598 (20.0)	< 0.001*
Long-term NIV, n(%)	4157	246 (5.9)	1171	83 (7.1)	2986	163 (5.5)	0.045*
Long-term CPAP, n(%)	4157	319 (7.7)	1171	68 (5.8)	2986	251 (8.4)	0.005*
Exacerbations last year (≥1 severes or ≥ 2 moderate), n (%)	3409	1251 (36.7)	1171	389 (33.2)	2238	862 (38.5)	0.002*
Cardio-vascular comorbidity	4157	2484 (59.8)	1171	690 (58.9)	2986	1794 (60.1)	0,494

Values were mean ± SD unless otherwise specified. * highlight statistically significant differences

Comparisons by Chi-square analysis (category variables) or T- test (normally distributed continuous variables) or Mann Whitney U test (not normally distributed continuous variables)

A category: no COPD treatment or short-acting bronchodilator(s) (SABA and/or SAMA) only; B category: LABA OR LAMA; C category: LABA+LAMA; D category: LABA OR LAMA + ICS; E category: LABA+LAMA+ICS

*HAD* hospital anxiety-depression scale, *mMRC* modified medical research council dyspnea scale, *CAT* COPD assessment test, *DIRECT* Disability Related to COPD Tool, *LTOT* long-term oxygen therapy, *NIV* non-invasive ventilation, *CPAP* continuous positive airway pressure

### Treatments

Treatments prescribed by respiratory physicians for each GOLD ABCD category are described in Table 2. Triple therapy (E) represented the most prescribed category (32.9%). Altogether, 29.8% of patients received a combination of two treatments, mostly two long-acting bronchodilators (18.5% vs 11.3% for ICS + LABA). Fifteen percent of the patients did not receive any inhaled treatment, 3.2% were prescribed short-acting agents only and 19% one long-acting bronchodilator. In more than half of cases, there was no short-acting bronchodilator on the prescription. Importantly, several discordances with current guidelines were identified: specifically many patients from A and B categories received an ICS (GOLD A: 24.5%, GOLD B: 37.4%), most patients were not prescribed any short-acting rescue bronchodilator (GOLD A: 84.6%, GOLD B: 70.4%, GOLD C: 82.1%, GOLD D: 48.0%), and some patients of B, C and D categories did not receive any long-acting bronchodilator (GOLD B: 18.1%, GOLD C: 32.1%, GOLD D: 10.8%).

**Table 2:** Description of inhaled treatments received by the analysed population, by GOLD ABCD category

	GOLD A	GOLD B	GOLD C	GOLD D	*p*-value
	*N* = 143	*N* = 676	*N* = 28	*N* = 324	
No bronchodilator	45 (31.5)	104 (15.4)	7 (25.0)	20 (6.2)	< 0.001*
SABA	22 (15.4)	199 (29.5)	5 (17.9)	167 (51.4)	< 0.001*
SAMA	3 (2.1)	60 (8.9)	3 (10.7)	78 (24.0)	< 0.001*
SABA or SAMA	22 (15.4)	200 (29.6)	5 (17.9)	169 (52.0)	< 0.001*
No SABA nor SAMA	121 (84.6)	475 (70.4)	23 (82.1)	156 (48.0)	< 0.001*
Only short-acting bronchodilators	3 (2.1)	18 (2.7)	2 (7.1)	15 (4.6)	0,159
Any LABA-containing regimen	72 (50.3)	446 (66.1)	17 (60.7)	276 (84.9)	< 0.001*
Any LAMA-containing regimen	66 (46.2)	436 (64.6)	11 (39.3)	235 (72.3)	< 0.001*
Any ICS-containing regimen	35 (24.5)	254 (37.6)	11 (39.3)	221 (68.0)	< 0.001*
LABA alone	16 (11.2)	51 (7.6)	3 (10.7)	7 (2.2)	< 0.001*
LAMA alone	23 (16.1)	107 (15.9)	2 (7.1)	14 (4.3)	< 0.001*
LABA+LAMA	21 (14.7)	143 (21.2)	3 (10.7)	50 (15.4)	0.05*
LABA+ICS	13 (9.1)	66 (9.8)	5 (17.9)	48 (14.8)	0,061
LAMA+ICS	0 (0.0)	0 (0.0)	0 (0.0)	0 (0.0)	–
LABA+LAMA+ICS	22 (15.4)	186 (27.6)	6 (21.4)	171 (52.6)	< 0.001*
Therapeutic category					
A	48 (33.6)	122 (18.1)	9 (32.1)	35 (10.8)	< 0.001*
B	39 (27.3)	158 (23.4)	5 (17.9)	21 (6.5)	< 0.001*
C	21 (14.7)	143 (21.2)	3 (10.7)	50 (15.4)	0,057
D	13 (9.1)	66 (9.8)	5 (17.9)	48 (14.8)	0,061
E	22 (15.4)	186 (27.6)	6 (21.4)	171 (52.6)	< 0.001*

Values are n (%). Treatment categories: A: no COPD treatment or short-acting bronchodilator(s) (SABA and/or SAMA) only; B: LABA OR LAMA; C: LABA+LAMA; D: LABA OR LAMA + ICS; E: LABA+LAMA+ICS. * highlight statistically significant differences

*SABA* short-acting beta2 agonist, *SAMA* short-acting antimuscarinic, *LABA* long-acting beta2 agonist, *LAMA* long-acting antimuscarinic, *ICS* inhaled corticosteroid

### Associations between patients’ characteristics and treatment patterns

Table 3 shows the main characteristics of the population depending on treatment categories. Analysing the effects of each possible predictor separately, it was found that treatment categories were influenced only by questionnaires scores and the severity of lung function impairment.

**Table 3:** Characteristics of patients by treatment category

Characteristics	Treatment category					*p*-value
	A	B	C	D	E	
	*N* = 214	*N* = 223	*N* = 217	*N* = 132	*N* = 385	
Age, in year	64.1 ± 8.4	65.6 ± 8.7	65.5 ± 8.1	65.6 ± 9.1	65.4 ± 8.2	0,444
Gender, male, n(%)	139 (65.0)	142 (63.7)	168 (77.4)	101 (76.5)	261 (67.8)	0.004*
BMI, kg/m^2^	26.0 ± 5.5	26.7 ± 5.8	26.1 ± 6.2	27.1 ± 6.1	25.5 ± 5.8	0,051
COPD GOLD stages, n(%)						
GOLD I	106 (49.5)	66 (29.6)	21 (9.7)	26 (19.7)	22 (5.7)	< 0.001*
GOLD II	82 (38.3)	124 (55.6)	106 (48.8)	58 (43.9)	131 (34.0)	< 0.001*
GOLD III	18 (8.4)	26 (11.7)	69 (31.8)	34 (25.8)	147 (38.2)	< 0.001*
GOLD IV	8 (3.7)	7 (3.1)	21 (9.7)	14 (10.6)	85 (22.1)	< 0.001*
FEV1, % predicted	77.0 ± 20.0	69.4 ± 17.5	55.8 ± 18.9	58.9 ± 21.5	47.4 ± 18.7	< 0.001*
FVC, % predicted	99.3 ± 21.1	93.5 ± 20.7	86.3 ± 22.5	85.1 ± 24.2	79.6 ± 22.9	< 0.001*
Active smoker, n(%)	111 (52.4)	93 (41.7)	73 (33.8)	54 (41.2)	118 (31.4)	< 0.001*
Cumulative smoking, pack-years	45.2 ± 22.3	43.7 ± 19.8	47.7 ± 23.9	45.4 ± 24.0	46.0 ± 23.6	0,558
MRC Score	1.2 ± 1.0	1.4 ± 0.9	1.8 ± 1.0	1.7 ± 1.2	2.1 ± 1.1	< 0.001*
MRC Score ≥ 2, n (%)	77 (36.0)	87 (39.0)	128 (59.0)	68 (51.5)	267 (69.4)	< 0.001*
HAD Score	11.4 ± 7.3	11.2 ± 6.6	11.7 ± 6.9	12.7 ± 6.9	13.6 ± 7.3	< 0.001*
CAT score	14.0 ± 7.2	14.8 ± 6.6	17.0 ± 7.3	17.8 ± 8.5	19.1 ± 7.6	< 0.001*
DIRECT score	8.4 ± 6.8	9.0 ± 5.7	12.2 ± 7.2	12.3 ± 8.0	14.9 ± 7.7	< 0.001*
LTOT, n(%)	19 (8.9)	6 (2.7)	30 (13.8)	14 (10.6)	101 (26.2)	< 0.001*
Long-term NIV, n(%)	14 (6.5)	20 (9.0)	14 (6.5)	8 (6.1)	27 (7.0)	0,801
Long-term CPAP, n(%)	9 (4.2)	4 (1.8)	10 (4.6)	8 (6.1)	37 (9.6)	< 0.001*
Exacerbations last year (≥1 severes or ≥ 2 moderate), n (%)	48 (22.4)	34 (15.2)	56 (25.8)	59 (44.7)	192 (49.9)	< 0.001*
Cardio-vascular comorbidity	117 (54.7)	142 (63.7)	123 (56.7)	80 (60.6)	228 (59.2)	0,367

Values were mean ± SD unless otherwise specified. * highlight statistically significant differences

A category: no COPD treatment or short-acting bronchodilator(s) (SABA and/or SAMA) only; B category: LABA OR LAMA; C category: LABA+LAMA; D category: LABA OR LAMA + ICS; E category: LABA+LAMA+ICS

*P*-value was obtained by performing Chi-square analysis (category variables) or ANOVA (normally distributed continuous variables) or Kruskal-Wallis Test (not normally distributed continuous variables)

*LTOT* long-term oxygen therapy, *NIV* non-invasive ventilation, *CPAP* continuous positive airway pressure, *HAD* hospital anxiety-depression scale, *CAT* COPD assessment, *mMRC* modified medical research council dyspnea scale

Table 4 sums up the predicting variables and the probability of prediction error of the different analyses. Only multiple logistic regression, penalized multiple regression and regression trees integrating more clinical characteristics including HAD and/or CAT and/or DIRECT are shown since general additive modelling completely supports the linearity effects (data not shown).

**Table 4:** Summary of multiple logistic regression analyses and regression trees. PPE: probability of prediction error

Analysis	Multiple logistic regression analyses		Penalized logistic regression analyses		Regression trees	
	Determinants	PPE	Determinants	PPE	Determinants	PPE
A vs BCDE (no maintenance vs at least one)	FEV1, FVC	0.19	FEV1, FVC, gender, age, CAT, DIRECT	0.17	FEV1, FVC	0.19
B vs CDE (one vs > 1 maintenance treatment)	FEV1, FVC, gender, exacerbation history	0.23	FEV1, FVC, gender, DIRECT, exacerbation history	0.22	FEV1, age, CAT, HAD, exacerbation history	0.20
BC vs DE (ICS vs no ICS in patients with a maintenance treatment)	FEV1, exacerbation history	0.34	FEV1, exacerbation history, age, gender, HAD, CAT, DIRECT	0.34	FEV1, FVC, age, HAD, exacerbation history	0.28
E vs BCD (triple vs other maintenance treatment schemes)	FEV1, FVC, exacerbation history	0.32	FEV1, gender, HAD, DIRECT, exacerbation history	0.32	FEV1, DIRECT, exacerbation history	0.29

A category: no COPD treatment or short-acting bronchodilator(s) (SABA and/or SAMA) only; B category: LABA OR LAMA; C category: LABA+LAMA; D category: LABA OR LAMA + ICS; E category: LABA+LAMA+ICS

Multiple logistic regression analyses provided the simplest models, which are detailed in Additional file 1: Tables S1-S4: in these models, lung function (FEV1 ± FVC) was associated with all treatment decisions; exacerbation history, gender and DIRECT score were associated with the decision to prescribe more than one vs only one maintenance treatment; exacerbation history was also associated with the decision to prescribe triple therapy vs all other maintenance options. More powerful predicting models as penalized logistic regressions and regression trees make emerge others determinant clinical predictors including HAD and/or CAT and/or DIRECT.

The regression trees select FEV1, exacerbation history and DIRECT score as predictive variables of treatment categories (A, B, C, D, E) (Additional file 1: Figure S1). The resulting misclassification rate was 0.51. The results of its application to uncategorized data is shown in Additional file 1: Figure S2; the identified thresholds were used to categorize variables.

**Fig. 1:**
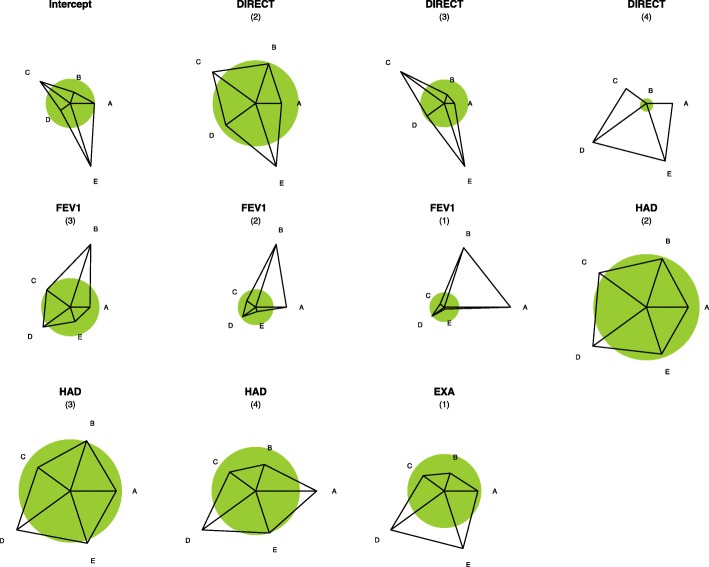
Effect Star Plots. Glyphs (effect star plots) shows the strength of associations between predictive variables and treatment categories. The star plot for each variable shows how strong the impact of the predictor on treatment choice is and what form it takes. The (shaded) unit circle around the center of each star corresponds to no-effect. A deviation from the circle shows the strength of the preference for one category as the deviation from the circle. If the ray is outside the circle, the increase in the predictor increases the probability of the corresponding category; if it is inside the circle, the increase in the predictor decreases the response probability. Stars are standardized such that the maximal length of a ray has the same value. This value also scales the radius of the unit circle. For example, consider the effect of the DIRECT variable: it is obvious that with increasing DIRECT, category E is more strongly favored while, in particular, the response probability for the A’s decreases. A category: no COPD treatment or short-acting bronchodilator(s) (SABA and/or SAMA) only; B category: LABA OR LAMA; C category: LABA+LAMA; D category: LABA OR LAMA + ICS; E category: LABA+LAMA+ICS

Associations between treatment categories and predictive variables (categorized and continuous) are presented using glyphs representations to facilitate understanding (Fig. 1) while a more complex mosaic representation is shown in Additional file 1: Figure S1.

Finally, repeating analyses with the mMRC score instead of or added to the DIRECT did not significantly change the PPE of the models (regression tree PPE: 0.53 and 0.49, respectively).

## Discussion

The aims of this study were to observe the frequency of inhaled treatment strategies in a large French COPD population, and to determine if specific clinical and-or functional factors were associated to prescription choices in real-life conditions. In this cohort of patients with all grades of airflow obstruction, the majority of patients (62.7%) received two or three maintenance agents. While lung function was associated with all treatment decisions, exacerbation history, CAT and DIRECT scores and, surprisingly, gender, were associated with the prescription of > 1 maintenance treatment. Several complementary and robust statistical models were used to expand simple multiple logistic regression for identifying factors explaining treatment choices. Results demonstrated that symptoms and respiratory function are clearly associated with escalating combinations of inhaled treatment but that the strength of associations remains relatively low despite clear French-language and global international recommendations [1, 21].

### Comparison with previous studies and interpretation of the results

The most striking feature in terms of overall treatment choices is the very high proportion of patients receiving multiple maintenance treatments. This is in line with previous studies in this field including those that recently focused on the use triple therapy [22, 23]. One explanation of this high use of multiple treatments is certainly the incomplete reversibility of COPD’s pathological and functional impairments: full control of both symptoms and exacerbations is infrequent, and patients most often keep some level of exertional dyspnoea. As a consequence, the treatment is frequently stepped up until no additional option is available. In addition, step-down treatment adaptations, although studied in a few trials, are not the topic and firm and clear recommendations [24, 25]. Reassuringly, multiple maintenance therapy was markedly less frequent in patients with grade 1 level of airflow limitation.

As in many other studies, the rate of ICS use is high in this cohort, although it does not exceed half of the population, contrary to what has been observed for many years by several previous studies in France [11, 26, 27] and elsewhere [12, 14, 22]. One explanation for this relatively lower ICS use in the present cohort might be that dual bronchodilation is recently recommended for exacerbation prevention in the majority of patients [1]. However, ICS were still markedly overused, as shown by the excessive proportion of GOLD A/B patients who received these agents, mostly as part of dual or triple associations with long-acting bronchodilators. Again, this is in line with most previous studies on this topic.

Triple therapy was prescribed in approximately half of patients with GOLD 3 and in the vast majority of patients with FEV1 less than 30% predicted. This apparent relation between treatment intensity and lung function was confirmed in all models used to identify the factors associated with therapeutic options. This contrasts markedly with the disappearance of FEV1 from treatment algorithms in virtually all recent guidelines. Although FEV1 is not a criterion guiding pharmacological treatment choice anymore, it has been used to guide ICS use during almost 2 decades. In addition, although FEV1 is a weaker predictor of exacerbation risk, lower FEV1 levels are associated with more future exacerbations independently of past exacerbation history [28].

Only a few studies assessed the factors associated with treatment choices in COPD. Recent analyses of the COPD gene cohort found that the intensity of treatment, as estimated using the total number of COPD medications, is associated with exacerbation rate as well as with gas trapping and airway wall thickness on CT-scan [29]. The importance of exacerbations as triggers of ICS prescription has been suggested in several other studies [14, 15, 30]. Many of these also identified associations between symptoms burden and treatment intensity [14, 23, 31]. In UK general practices, ICS use in GOLD A/B patients (in whom there is theoretically no indication of ICS according to most guidelines) appears associated with the level of airflow obstruction, concurrent asthma diagnosis and exacerbation rates as well as the region of the practice [30], suggesting that although markers of disease severity play a role, less objective or “scientific” factors are also involved. Other less understandable features such as gender have also been identified as associated with the use of some treatment schemes, i.e., triple therapy [31]. Previous studies used the % of explained variance to quantify how treatments are associated with patients’ characteristics [26], and found very low figures. Here the misclassification rate, which illustrates to which extent clinical features can explain treatment choices, is more encouraging. However, these two methods are not really comparable.

In our study, prescribers were exclusively pulmonary physicians. Interestingly, once initiated maintenance COPD treatments are infrequently modified, determinants of changes being again dominated by exacerbations and symptoms [14]. Most often, treatment profiles do not differ markedly between specialists’ and GPs’ prescriptions [32]. Importantly, all the treatments considered in the study are equally reimbursed in France, with no major disproportion in their costs. As a consequence, an economic influence on treatment decisions is unlikely.

### Strengths of the statistical strategy

One highly original feature of the current study is the use of several complementary parametric and nonparametric statistical regression techniques with multiple graphical approaches to express observed relations. To our knowledge, this is the first study to use such an approach, which optimises the robustness of results and increases the chance of better deciphering factors associated with treatment choices. Notably, this novel approach permits the calculation of the prediction error for all tested models, allowing to determine which of them is the most reliable to identify possible determinants of treatment choices, and what is the magnitude of the difference in reliability between models. Importantly, treatments were categorised based on current international guidelines to facilitate analyses and interpretation of results. An in-depth discussion of the analytical strategy can be found in the electronic supplement. Altogether, we believe this rather complex approach is of high interest to better understand the basis of clinical reasoning while making treatment choices.

### Limitations and implications for future research

One limitation of this study is that the studied sample cannot be considered as fully representative of the French population of patients with COPD, for several reasons. Firstly, investigators were clinicians agreeing to participate instead of a random sample of the French population of respiratory Physicians. Accordingly, there was a marked imbalance between hospital-based (78%) and private practitioners (22%, *n* = 32), and we cannot exclude an influence of the type of practice on treatment decisions. However, given the number of individual treatment options and strategies of interest, it was decided to refrain from analysing their relation with the type of practice since the results would not be robust and could be misleading. Secondly, a high proportion of the cohort’s population could not be studied since all required data were not available. However, even if some differences between patients with vs without complete data were statistically significant, they were of marginal clinical significance. A more extensive characterization of patients could have revealed other potential determinants of treatment choices, but corresponding data (e.g., bronchodilator reversibility, blood eosinophil count or lung volumes) were not available in many patients, preventing from integrating them in the analyses. Similarly, the study design did not allow to assess the link between adherence and inhalation technique on the one hand, and outcomes (e.g., exacerbations) on the other. As a consequence of the above-mentioned limitations, it will be important to further test the generalisability of the present findings. The methods and results reported here should form a useful basis in that respect.

During the last decades, the evidence base on pharmacological options for COPD treatment has increased considerably. In parallel, every year many guidelines on COPD are produced at various levels (national, regional or global) [7]. Although recent innovations relate more to inhalation devices and treatment associations within the same device, there has been a multiplication of available therapeutic solutions. The high proportion of potential deviations from guidelines that was observed here needs to be considered carefully since we don’t have access to the historical sequence between treatment choices and clinical characteristics, which represents a limitation inherent to the cross-sectional nature of the study. In addition, the main purpose of the present analysis was to determine whether physicians follow clinical features when choosing treatments, which appeared to be the case, but not whether this rationale is the same as in guidelines, which does not seem to be completely the case. This issue cannot be explored further since physicians are not asked to provide the rationale of their choices in the COLOBIRI platform. Regarding short-acting bronchodilators, we cannot rule out a parallel prescription by the patients’ general practitioners. However, many practice surveys in various countries also found that guidelines remain poorly implemented, and potential barriers to implementation need to be better elucidated [16]. Understanding factors associated with treatment decisions may allow to develop more targeted strategies to improve guidelines’ implementation in routine care. In addition, it may guide the design of real-life effectiveness research studies to maximise the relevance of results in routine patient care.

## Conclusion

This real-life cohort study found that most COPD patients cared for by pulmonary physicians in France receive multiple maintenance treatments, frequently including ICS although their indications are limited to specific populations in recent guidelines. Lung function, exacerbation history and symptoms burden assessed by questionnaires are among the most important factors associated with treatment choices. Still, an important part of treatment choices is not associated with clinical presentation. Thus, rationalising treatment choices is a crucial goal for upcoming guidelines and should be helped by improved real-life effectiveness studies as well as a better understanding of barriers to guidelines implementation and real-life drivers of therapeutic strategies.

## Additional file

Additional file 1: **Figure S1.** Mosaics showing associations between categorized variables and treatments. **Figure S2.** Example of a Regression Tree developed in the multinomial model with treatment category (B, C, D versus E) as explained variable. **Table S1.** Multiple logistic regression analysis of no vs at least one inhaled maintenance therapy. **Table S2.** Multiple logistic regression analysis of one vs more than one maintenance treatment. **Table S3.** Multiple logistic regression analysis of maintenance treatment without vs with ICS. **Table S4.** Multiple logistic regression analysis of triple therapy vs all other maintenance treatment options. (DOCX 120 kb)

## Data Availability

The datasets used and/or analysed during the current study are available from the corresponding author on reasonable request.

## Abbreviations

CAT: COPD assessment test
CPAP: Continuous positive airway pressure
DIRECT: Disability Related to COPD Tool
HAD: Hospital anxiety-depression scale
ICS: Inhaled corticosteroid
LABA: Long-acting beta2 agonist
LAMA: Long-acting antimuscarinic agent
LTOT: Long-term oxygen therapy
mMRC: modified medical research council dyspnea scale
NIV: Non-invasive ventilation
SABA: Short-acting beta2 agonist
SAMA: Short-acting antimuscarinic agent
