# Supplementary material for: Are there specific clinical characteristics associated with physician’s treatment choices in COPD?
Source: Respir Res. 2019 Aug 20;20:189. doi: 10.1186/s12931-019-1156-1 (PMC6701115; doi:10.1186/s12931-019-1156-1)
Supplement: Supplementary file 1 — Figure S1. Mosaics showing associations between categorized variables and treatments. Figure S2. Example of a Regression Tree developed in the multinomial model with treatment category (B, C, D versus E) as explained variable. Table S1. Multiple logistic regression analysis of no vs at least one inhaled maintenance therapy. Table S2. Multiple logistic regression analysis of one vs more than one maintenance treatment. Table S3. Multiple logistic regression analysis of maintenance treatment without vs with ICS. Table S4. Multiple logistic regression analysis of triple therapy vs all other maintenance treatment options. (DOCX 120 kb) [file 12931_2019_1156_MOESM1_ESM.docx]

**ELECTRONIC SUPPLEMENT**

**Determinants of treatment choices in COPD: what is the weight of clinical presentation?**

Nicolas Roche^1^, Anestis Antoniadis^2^, David Hess^3^, Pei Zhi Li^4^, Eric Kelkel^5^, Sylvie Leroy^6^, Christophe Pison^7^, Pierre-Régis Burgel^8^ and Bernard Aguilaniu^9^

**Justification and detailed description of the analytical strategy**

*First phase of the analyses*

Following recoding of patients’ prescribed treatment as dichotomous responses, recoded responses were analysed using standard multiple logistic regression. In general, the most relevant explanatory covariates are selected using stepwise processes. Here, to avoid over fitting we have also used penalized multiple logistic regression with LASSO regularization as it is implemented in the R-package glmnet (the amount of regularization is adjusted by cross validation)[19]. Indeed, the multiple logistic regression model is restricted in the sense that all variables affect the logit in a strictly linear fashion. To further check that the effect of continuous explanatory variables is linear in predicting the probability of the response we have also used non-parametric logistic regression as it is implemented in the R-package mgcv (function gam). Finally since logistic models are susceptible to multicollinearity (i.e., high correlations between predictors) we have also used a nonparametric technique based on an evolutionary algorithm for learning globally optimal classification and regression trees (implemented in the R-package evtree) which is known to be resistant to highly correlated predictors.

*Second phase of the analyses*

The use of parameter glyphs was triggered by two characteristics of multinomial multiple logit regression for nominal multi-category responses: first, many parameters are involved in such models, and second, interpretation of parameters is much harder than for linear models because the model is nonlinear. Both issues can profit from graphical representations.

*Third phase of the analyses*

When the data is categorical (both response and predictors are qualitative) graphical methods for the analysis of multiway contingency tables in the form of mosaic plots [2] are widely used (see supplementary Figure 1). Mosaic displays are essentially graphical methods for displaying pairwise associations views of categorical data. When some of the significant variables are quantitative, they may be transformed into categorical ones using, for example, their estimated splits from a regression tree fitted model (see supplementary figure 2 A), allowing therefore to view the data as categorical and visualizing the resulting contingency table via a mosaic plot.

**Discussion of the analytical strategy**

Among the parametric approaches, we have first used a one-vs-rest scheme to explore factors that are associated to each treatment choice using separate binary logistic regression models. However, although such an approach is useful, given that (1) the response has more than two categories and (2) our aim is to compare the influence of all observed predictors on all the treatment choices, we have also adopted a polytomous or multinomial logistic regression model, which seems to be more efficient. To also address an eventual collinearity among the predictors, to avoid overfitting and to perform simultaneously variable selection, we have used a penalised version of the above fitting procedures. One problem with the multinomial logit model is that many parameters are involved; another is that interpretation of parameters is much harder than for linear models because the model is non-linear. We have therefore used star plots to visualize the predictor’s effects strengths and consolidate our findings. To analyse further the results, we have also used an alternative nonparametric approach, fitting a regression tree to the data and used the fitted tree to predict the response. A main advantage of an evolutional regression tree as compared with polytomous logistic regression is its much higher prediction accuracy. Moreover, a regression tree can select important variables automatically no matter how many variables are used initially and whatever their degree of correlation is. The algorithm which a tree uses to select important variables is done simultaneously within the fitting procedure and takes into account eventual interactions between the predictors and is therefore different from classical stepwise variable selection procedures used in logistic regression.

Supplementary references:

1. Tutz G, Schauberger G. Visualization of Categorical Response Models: From Data Glyphs to Parameter Glyphs. *J. Comput. Graph. Stat.* 2013; 22: 156–177.

2. Friendly M. Mosaic Displays for Multi-Way Contingency Tables. *J. Am. Stat. Assoc.* 1994; 89: 190–200.

Supplementary figure 1: Mosaics showing associations between categorized variables and treatments

The mosaic display is a graphical method for visualizing an n-way contingency table and for building models to account for the associations among its variables. The frequencies in a contingency table are portrayed as a collection of rectangular “tiles” whose areas are proportional to the cell frequencies; the areas are colored and shaded to portray the residuals from a specified log-linear model. Blue rectangles correspond to high positive correlations, red ones to high negative correlations and grey ones to low or no correlations. The mosaic display reveals the pattern of lack of fit.

Once one has the predicted probabilities from the fitted logistic model, a threshold needs to be chosen to classify the observations. The choice will depend on whether the purpose is to optimize sensitivity, specificity or whatever measure is most important in the context of the application. In our case we have used .5 as a cutoff point which seems to give the highest prediction in our model.

Supplementary Figure 2: Example of a Regression Tree developed in the multinomial model with treatment category (B,C,D versus E) as explained variable.


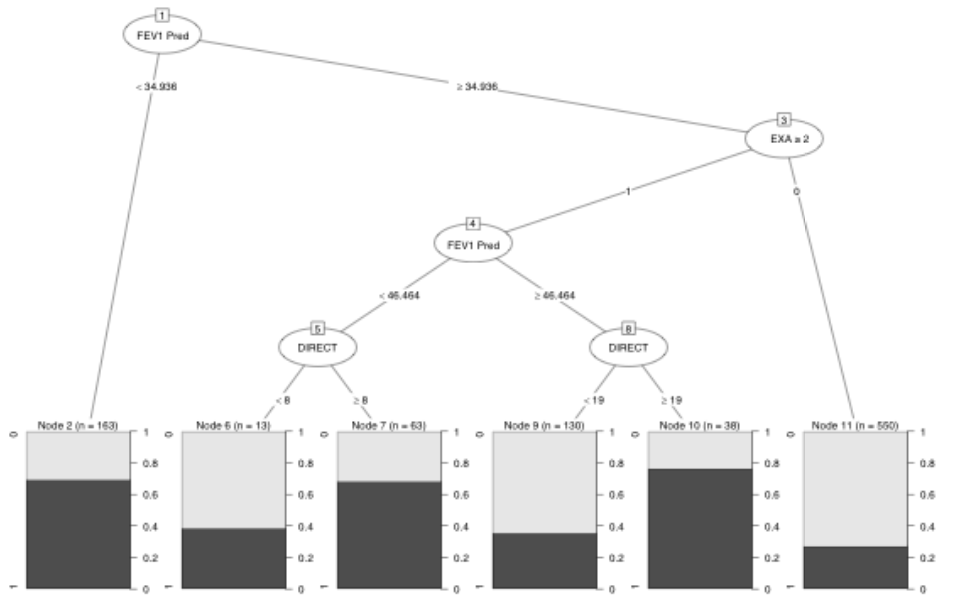


A fitted regression tree to the training data (excluding patients of category A) to model the regression of the binary variable (coded 0 for a patient with treatment B or C or D and taking the value 1 for a patient with treatment E) with respect to the set of all predictors. The resulting selected variables are FEV1 %pred, DIRECT and EXA≥ 2. Each terminal node shows the proportions of patients within the node that had treatment E (black) vs the proportion of patients not having E (gray). These proportions allow to predict the treatment of any new patient in the testing data sample.

Supplementary table E1: multiple logistic regression analysis of no vs at least one inhaled maintenance therapy

|  | Estimate | Std. Error | z value | Pr(>\|z\|) |
| --- | --- | --- | --- | --- |
| Intercept | -2.262520 | 0.845622 | -2.676 | <0.01 |
| AGE | -0.013885 | 0.010099 | -1.375 | 0.17 |
| HAD | 0.013863 | 0.014496 | 0.956 | 0.34 |
| CAT | -0.026395 | 0.016338 | -1.616 | 0.11 |
| DIRECT | -0.028690 | 0.018071 | -1.588 | 0.11 |
| FEV1 | 0.060059 | 0.007165 | 8.382 | <0.001* |
| FVC | -0.018739 | 0.006408 | -2.924 | <0.01* |
| Male vs female | -0.151631 | 0.185512 | -0.817 | 0.41 |
| EXA2 | 0.025173 | 0.202592 | 0.124 | 0.90 |

Supplementary table E2: multiple logistic regression analysis of one vs more than one maintenance treatment

|  | Estimate | Std. Error | z value | Pr(>\|z\|) |
| --- | --- | --- | --- | --- |
| Intercept | -2.488920 | 0.875674 | -2.842 | <0.01 |
| AGE | 0.006786 | 0.010527 | 0.645 | 0.52 |
| HAD | -0.002301 | 0.014962 | -0.154 | 0.88 |
| CAT | -0.002420 | 0.016351 | -0.148 | 0.88 |
| DIRECT | -0.054325 | 0.019018 | -2.857 | <0.01* |
| FEV1 | 0.053597 | 0.007210 | 7.434 | <0.001* |
| FVC | -0.023806 | 0.006328 | -3.762 | <0.01* |
| Male vs female | -0.632796 | 0.194175 | -3.259 | <0.01* |
| EXA2 | 1.063478 | 0.220621 | 4.820 | <0.001* |

Supplementary table E3: multiple logistic regression analysis of maintenance treatment without vs with ICS

|  | Estimate | Std. Error | z value | Pr(>\|z\|) |
| --- | --- | --- | --- | --- |
| Intercept | -1.1635536 | 0.7033842 | -1.654 | 0.10 |
| AGE | -0.0004668 | 0.0086527 | -0.054 | 0.96 |
| HAD | -0.0140739 | 0.0118837 | -1.184 | 0.24 |
| CAT | -0.0042223 | 0.0133338 | -0.317 | 0.75 |
| DIRECT | -0.0219990 | 0.0146904 | -1.498 | 0.13 |
| FEV1 | 0.0276699 | 0.0054172 | 5.108 | <0.001* |
| FVC | -0.0072331 | 0.0046871 | -1.543 | 0.12 |
| Male vs female | -0.1080918 | 0.1620360 | -0.667 | 0.50 |
| EXA2 | 1.0195040 | 0.1576335 | 6.468 | <0.001* |

Supplementary table E4: multiple logistic regression analysis of triple therapy vs all other maintenance treatment options

|  | Estimate | Std. Error | z value | Pr(>\|z\|) |
| --- | --- | --- | --- | --- |
| (Intercept) | 0.755952 | 0.709686 | 1.065 | 0.29 |
| AGE | 0.001398 | 0.008811 | 0.159 | 0.87 |
| HAD | 0.010933 | 0.011930 | 0.916 | 0.36 |
| CAT | -0.004463 | 0.013547 | -0.329 | 0.74 |
| DIRECT | 0.028186 | 0.014722 | 1.915 | 0.06 |
| FEV1 | -0.041014 | 0.005724 | -7.166 | <0.001* |
| FVC | 0.013108 | 0.004767 | 2.750 | <0.01* |
| Male vs female | -0.162910 | 0.163742 | -0.995 | 0.32 |
| EXA2 | -0.663762 | 0.153793 | -4.316 | <0.001* |
